# Deciphering CD59: Unveiling Its Role in Immune Microenvironment and Prognostic Significance

**DOI:** 10.3390/cancers16213699

**Published:** 2024-11-01

**Authors:** Bhaumik Patel, Ashok Silwal, Mohamed Ashraf Eltokhy, Shreyas Gaikwad, Marina Curcic, Jalpa Patel, Sahdeo Prasad

**Affiliations:** 1 Department of Immunotherapeutic and Biotechnology, Texas Tech University Health Science Center, Abilene, TX 79601, USA; bhaumik.patel@ttuhsc.edu (B.P.); ashok.silwal@ttuhsc.edu (A.S.); mohamed.eltokhy@ttuhsc.edu (M.A.E.); shreyas.gaikwad@ttuhsc.edu (S.G.); mcurcic@ttuhsc.edu (M.C.); jalpa.patel@ttuhsc.edu (J.P.); 2 Masonic Cancer Center, University of Minnesota, Minneapolis, MN 55455, USA

**Keywords:** CD59, complements, immune suppression, overall survival, immune evasion

## Abstract

**Simple Summary:**

CD59 prevents the formation of membrane attack complex, enabling tumor cells to escape complement-mediated cytotoxicity. It appears that the expression of CD59 significantly impacts survival outcomes due to its interaction with immune cells. The associations between CD59 and immune suppressive cells such as T-regulatory cells, myeloid derived suppressor cells, and macrophage create an immune suppressive environment in cervical, brain, head and neck, and stomach cancers. However, kidney cancer has less or no association, leading to better survival outcomes. Thus, assessing the levels of CD59 and its immune cell associations is crucial as it plays a predictive role in deciding prognostic outcomes.

**Abstract:**

Background: CD59, a GPI-anchored membrane protein, protects cancer cells from complement-dependent cytotoxicity (CDC) by inhibiting the formation of the membrane attack complex (MAC). It has been demonstrated to be overexpressed in most solid tumors, where it facilitates tumor cell escape from complement surveillance. The role of CD59 in cancer growth and interactions between CD59 and immune cells that modulate immune evasion has not been well explored. Methods: Using cancer patient database from The Cancer Genome Atlas (TCGA) and other public databases, we analyzed CD59 expression, its prognostic significance, and its association with immune cell infiltration in the tumor microenvironment, identifying associated genomic and functional networks and validating findings with invitro cell-line experimental data. Results: This article describes the abundant expression of CD59 in multiple tumors such as cervical squamous cell carcinoma (CESC), kidney renal cell carcinoma (KIRC), glioblastoma multiforme (GBM), head and neck squamous cell carcinoma (HNSC), and stomach adenocarcinoma (STAD), as well as in pan-cancer, using The Cancer Genome Atlas (TCGA) database and confirmed using multiple cancer cell lines. The expression of CD59 significantly alters the overall survival (OS) of patients with multiple malignancies such as CESC, GBM, HNSC, and STAD. Further, the correlation between CD59 and Treg and/or MDSC in the tumor microenvironment (TME) has shown to be strongly associated with poor outcomes in CESC, GBM, HNSC, and STAD as these tumors express high FOXP3 compared to KIRC. Moreover, unfavorable outcomes were strongly associated with the expression of CD59 and M2 tumor-associated macrophage infiltration in the TME via the IL10/pSTAT3 pathway in CESC and GBM but not in KIRC. In addition, TGFβ1-dominant cancers such as CESC, GBM, and HNSC showed a high correlation between CD59 and TGFβ1, leading to suppression of cytotoxic T cell activity. Conclusion: Overall, the correlation between CD59 and immune cells predicts its prognosis as unfavorable in CESC, GBM, HNSC, and STAD while being favorable in KIRC.

## 1. Introduction

CD59 is a GPI-anchored membrane protein (mCD59) that plays a crucial role in regulating the complement system, which is an essential component of the innate system. CD59 is primarily found throughout the body as a regulator of cell lysis through the complement system on erythrocytes, leukocytes, fibroblasts, and various epithelial cell surfaces, including the pancreas, epidermis, bronchi, kidney, and salivary glands, where it acts as a potent inhibitor of the complement cascade, preventing the excessive activation and damage of host cells [1,2,3]. CD59 achieves its inhibitory function by binding to complement proteins C8 and C9 and preventing their assembly into the membrane attack complex (MAC). The MAC is responsible for creating pores in the membranes of target cells, leading to their destruction. By inhibiting MAC formation, CD59 helps to maintain the integrity and survival of host cells, protecting them from complement-mediated lysis [4].

In addition, CD59 also interacts with linker for activation of T cells (LAT) by transferring a palmitate group to LAT, causing them to co-localize in the lipid rafts. This interaction regulates T cell signal transduction [5]. Gallimore et al. demonstrated that upregulation of CD59 on activated CD4+ T cells serves to down-regulate their activity in response to polyclonal and antigen-specific stimulation. This indicates that CD59 impacts on human CD4+ T cells and determines whether or not their blockade boosts immune responses [6].

Deficiencies or abnormalities in CD59 expression or function can have serious implications on the immune system and overall health. Inadequate CD59 activity can result in excessive complement activation, leading to various known disorders, including paroxysmal nocturnal hemoglobinuria (PNH), an acquired hematological disorder characterized by the destruction of red blood cells [7].

CD59 is widely expressed, and its expression levels are higher in the majority of cancer cells than in adjacent normal cells [8]. In most cancers, CD59 acts as a defense mechanism utilized by tumor cells to evade destruction by the immune system. The ability of CD59 to inhibit MAC formation and downregulate CD4+ T cells plays an important role in the suppression of tumor immune environments. However, the expression of CD59 in human cancer and its impact on prognosis and targeted therapy remains under-explored. Therefore, in this article, we have investigated the expression, prognostic significance, and impact on immune cell infiltration in the tumor microenvironment using CD59 expressive cancer patient data in The Cancer Genome Atlas (TCGA) and various publicly available databases. Using a variety of analysis methods, we identified the genomic and functional networks associated with the expression of CD59 in human cancers. Generated meta-analysis data were confirmed using the cell-line approach. Thus, our findings reveal new insight into CD59 and its immune interactions, which can be utilized for human cancer diagnosis, prognosis, and treatment.

## 2. Materials and Methods

### 2.1. ShinyGO v0.741

To assess gene ontology enrichment analysis, we have used ShinyGo v0.741 (http://bioinformatics.sdstate.edu/go74/, accessed on 18 April 2024). ShinyGo biological process charts provide fold enrichment charts of the pathways affected by the expression of a particular gene. Fold enrichment is defined as the percentage of genes in the list belonging to a pathway, divided by the corresponding percentage in the background. Fold enrichment indicates how drastically genes of a certain pathway are overrepresented.

### 2.2. GEPIA Dataset

The expression of CD59 in normal and cancer tissues is analyzed using the GEPIA 2 (http://gepia2.cancer-pku.cn/, accessed on 12 April 2024) dataset. GEPIA is a web server for analyzing the RNA sequencing expression of a large number of tumors and normal samples from the TCGA data portal and the GTEx database, respectively. The single gene module of GEPIA was used to study the mRNA expression levels of CD59 in cancer tissues and normal tissues. Kaplan–Meier curves were used to study the survival outcomes, including disease-free survival (DFS) and overall survival (OS). Patients were divided into high and low CD59 groups according to the median transcripts per million (TPM) expressions.

### 2.3. Human Protein Atlas (HPA)

Protein expression of CD59 in normal and multiple cancers was checked by the Human Protein Atlas (HPA). Immunohistochemically stained images of CD59 expression in multiple cancers were collected from the HPA.

### 2.4. TISIDB

Tumor-immune system interactions and the drug bank database (TISIDB) (http://cis.hku.hk/TISIDB/, accessed on 17 April 2024), an integrated repository portal for tumor-immune system interactions, has been utilized to determine the correlation between Cd59 and lymphocytes, immunomodulators, and chemokines. TISIDB was also used to determine the association between CD59 and immune subtypes across multiple human cancers. The spearman correlation test was checked to determine significance.

### 2.5. TIMER

We analyzed the TCGA database using the Tumor Immune Estimation Resource site (TIMER) (http://timer.cistrome.org/, version 2.0, accessed on 1 April 2024) to check the correlation between CD59 and immune infiltrates for the systemic analysis of tumor-infiltrating immune cells in multiple tumors. In TIMER, the algorithm predicts the immune properties of tumors, and the genes module was used to assess the relationship between CD59 and immune cell infiltrates (CD8+ T cells, CD4+ T cells, B cells, macrophages, neutrophils, and dendritic cells). Spearman correlation was used for analysis, with a *p*-value < 0.05 considered significant.

### 2.6. Immunoblotting

HEK293T, 786-O, HeLa, and SF188 cells were lysed using RIPA. Protease and phosphatase inhibitor cocktails were added to 1× lysis buffer. Lysates were subjected to electrophoresis and probed for CD59 (Thermo Fisher Scientific# PA5-78993, Carlsbad, CA, USA), pSTAT3 (Invitrogen#44380G, Carlsbad, CA, USA), STAT3 (Cell Signaling#12640S, Danvers, MA, USA), TGFβ1(Invitrogen#MA121595, Carlsbad, CA, USA), β-tubulin (Cell Signaling#2146 Danvers, MA, USA), and β-actin (Cell Signaling #4970, Danvers, MA, USA) and their respective secondary HRP antibodies.

### 2.7. Immunofluorescence

Cells were fixed with 4% PFA in PBS and then blocked with 5% goat serum in PBS. CD59 (Thermo Fisher Scientific# PA5-78993) primary antibody was diluted in 1% BSA in PBS (antibody dilution buffer). Secondary antibody conjugated with fluorochrome (AF488) and DAPI-gel were used, and images were captured with a Nikon AXR confocal microscope (Nikon Corporation, Melville, NY, USA) and analyzed using Nikon Elements Advanced Research (NIS-Elements C Ver 6.02.03) software.

### 2.8. qRT-PCR

The q(RT)-PCR was performed using a High-Capacity cDNA Synthesis Kit (Applied Biosystems, cat# 4368814, Waltham, MA, USA), Fast power up SybrGreen (Applied Biosystems, cat# A25742, Waltham, MA, USA), and Step One Plus Applied Biosystems (Applied Biosystems, Waltham, MA, USA). Relative expression was calculated using the 2−ΔΔCt method and RT2 profiler PCR Array Data Analysis (SAB Biosciences, Miami Beach, FL, USA) and normalized to GAPDH. The following primers were used:

CD59: F-5′-TTTTGATGCGTGTCTCATTACCA-3′

R-5′-ATTTTCCCTCAAGCGGGTTGT-3′

GAPDH: F-5′-GGCTCTCCAGAACATCATCC-3′

R- 5′-ACTGACACGTTGGCAGTGG-3

IL10: F’-GACTTTAAGGGTTACCTGGGTTG-3′

R-5′-TCACATGCGCCTTGATGTCTG

### 2.9. Flow Cytometry

Flow cytometric surface and intracellular staining were performed using the antibody manufacturer protocol (BioLegend protocol) for PE/Cyanine7 anti-human LAP (TGF-β1) antibody (cat# 349610, BioLegend. Inc., San Diego, CA, USA), AF647 anti-human FOXP3 antibody (cat# 320113, BioLegend. Inc., San Diego, CA, USA), PerCP Cy5.5 anti- F/480 antibody (cat# 123127, BioLegend. Inc., San Diego, CA, USA), and PE anti-mouse CD206 antibody (cat# 162503, BioLegend. Inc., San Diego, CA, USA). For intracellular staining, FOXP3 Fix/perm Buffer (cat# 421403, BioLegend Inc., San Diego, CA, USA) was used, following the manufacturer’s protocol.

### 2.10. ELISA Assay

ELISA assay was performed using the manufacturer’s protocol. TGFB1 ELISA kits (Cat# 88-50390-22, Carlsbad, CA, USA) and IL10 ELISA kits (Cat#BMS215HS, Carlsbad, CA, USA) were purchased from Thermo Fisher Scientific.

### 2.11. Data Availability

The data used to support the findings of this study are publicly available. These data can be found in The Cancer Genome Atlas (TCGA), The Common Fund’s Genotype-Tissue Expression (GTEx), The University of Alabama at Birmingham (UALCAN), and Search Tool for the Retrieval of Interacting Genes/protein (STRING) databases.

### 2.12. Statistics

Data are presented as mean ± SEM. Differences between groups were evaluated using either a two-tailed or a one-tailed Student’s *t*-test. Statistical analyses were performed using GraphPad Prism, version 10.2.3, and *p*-values < 0.05 were considered statistically significant.

## 3. Results

### 3.1. Role of CD59 and Its Expression in Different Cancers

**Figure 1:**
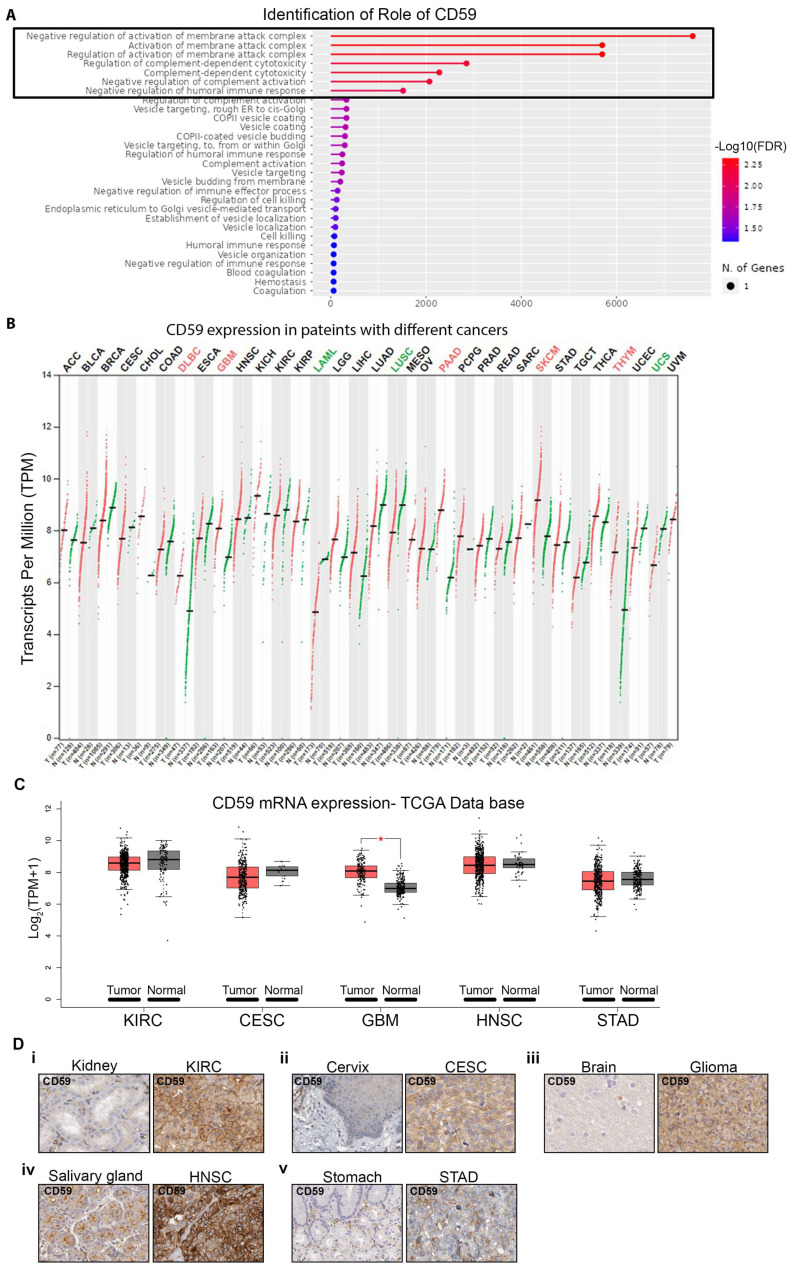
Role of CD59 and expression in different cancers. (**A**) involvement of CD59 in different biological pathways using ShinyGO v0.741. (**B**) Comparison of CD59 mRNA expression between cancer and its normal tissue counterpart in multiple cancers using the TCGA database and the GAPIA2 analytical tool. (**C**) mRNA expression of CD59 in KIRC, CESC, GBM, HNSC, and STAD cancer patients and normal tissue from the TCGA database using the GAPIA2 analytical tool. (**Di**–**v**) Protein expression of CD59 in KIRC (**i**), CESC (**ii**), GBM (**iii**), HNSC (**iv**), and STAD (**v**) cancer patients and normal tissue from the Human Protein Atlas. Each dot represents mRNA expression of sample and * indicates *p* ≤ 0.05.

The ShinyGO v0.741 gene ontology enrichment analysis of CD59 (Ensembl: ENSG00000085063) reveals that CD59 negatively regulates the activation of the membrane attack complex (MAC). This MAC formation is responsible for creating pores on targeted cells and regulates complement-mediated cytotoxicity. This functional role of CD59 makes it a key player in the negative regulation of complement-dependent cytotoxicity and a negative regulator of humoral immune response (Figure 1A). STRING analysis identifies genes significantly related to CD59 by analyzing the Kyoto Encyclopedia of Genes and Genomes (KEGG) (Figure S1A). The ShinyGO analysis of CD59 provides three aspects: cellular composition (CC), biological process (BP), and molecular function (MF). These data suggest that CD59 is mainly distributed in cellular membranes, and its main biological functions include regulating the activation of complements by limiting membrane attack complex formation and natural killer cell-mediated cytotoxicity (Figure S1B,C). Its molecular functions are related to protein binding (Figure S1B). The top 10 KEGG pathways are illustrated in Table 1. The GEPIA database provides a comparison of CD59 transcriptional levels in a variety of TCGA cancer samples with normal samples. CD59 mRNA transcription analysis reveals that multiple cancers, such as diffuse large B cell lymphoma (DLBC), glioblastoma multiforme (GBM), pancreatic adenocarcinoma (PAAD), skin cutaneous melanoma (SKCM), and thymoma (THYM) exhibit significantly higher mRNA expression of CD59 (Figure 1B). Overall, CD59 shows high mRNA expression in 14 out of 29 different cancer types (Figure 1B).

To investigate further, we analyzed the TCGA database and found that KIRC, CESC, GBM, HNSC, and STAD not only exhibit high mRNA expression (Figure 1C) but also have higher CD59 protein levels compared to their normal counterparts (Figure 1D). Additionally, the HPA database indicates that cancers like cervical, GBM, head and neck, melanoma, pancreatic, renal, and stomach cancers exhibit elevated CD59 expression, which significantly affects their prognosis (Table 2).

### 3.2. Abundant Expression of CD59 in Multiple Cancer Cell Lines

**Figure 2:**
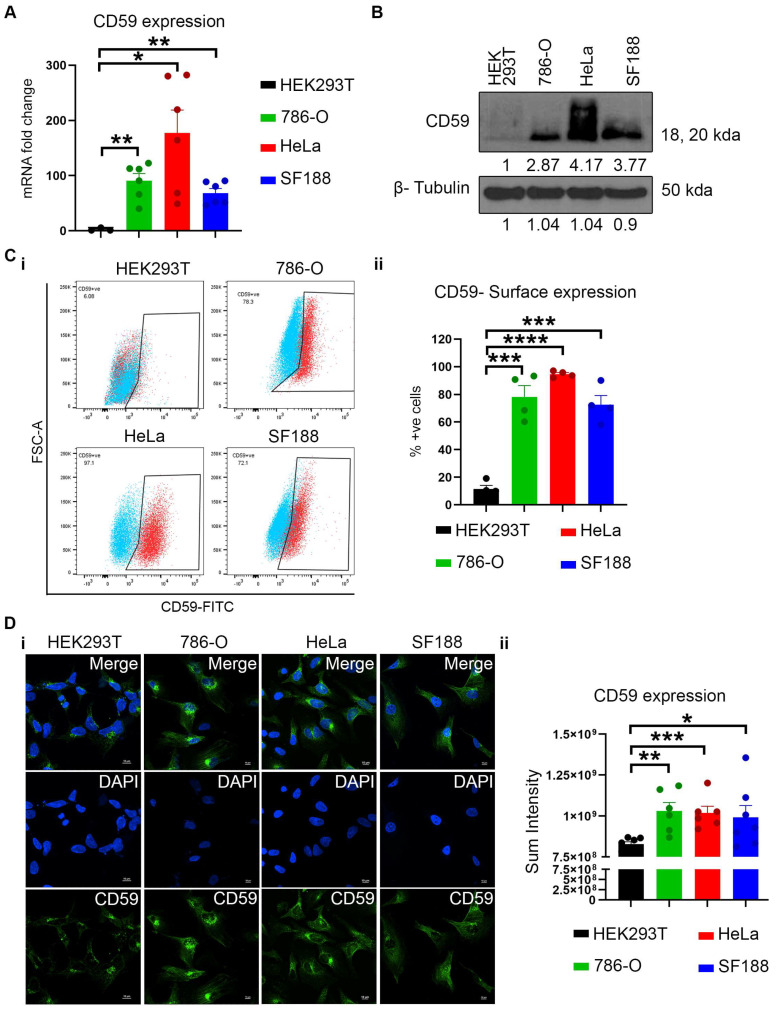
Expression of CD59 in cancer cell lines and cancer patients. (**A**) mRNA expression of CD59 in HEK 293T (normal human embryonic kidney), 786-O (KIRC), HeLa (CESC), and SF188 (GBM) by RT-PCR. (**B**–**D**) protein expression of CD59 in HEK 293T (normal human embryonic kidney), 786-O (KIRC), HeLa (CESC), and SF188 (GBM) by Western blot (**B**), flowcytometry (surface staining) (**Ci**), and its quantification (**Cii**), and immunofluorescence (surface staining) (**Di**) with its quantification (**Dii**). β-tubulin (control) for Figure 2B (786-O, HeLa, and SF188) are similar to Figure 7A(iii). Data represents the minimum of three independent experiments, where * indicates *p* ≤ 0.05, ** indicates *p* ≤ 0.01, *** indicates *p* ≤ 0.001, **** indicates *p* ≤ 0.0001, *t*-test.

The expression of CD59 at both the mRNA and protein levels has been assessed using various methods across multiple cell lines. RT-qPCR analysis of CD59 reveals that 786-O (represents KIRC), HeLa (represents CESC), and SF188 (represents GBM) express significantly higher mRNA of CD59 compared to HEK293T (represents normal kidney) (Figure 2A). Additionally, immunoblotting, flow cytometry, and immunofluorescence analysis reveal high protein expression in all tumor cell lines, with HeLa (CESC) exhibiting the highest expression among them compared to normal kidney (Figure 2B–D). These data confirm the finding that most cancers increase CD59 expression to fight against the immune system. Taken together, this evidence indicates that high CD59 levels may play a key role in immune escape, leading to tumor development and poor prognosis.

### 3.3. Prognostic and Diagnostic Value of CD59 in Multiple Cancer

**Figure 3:**
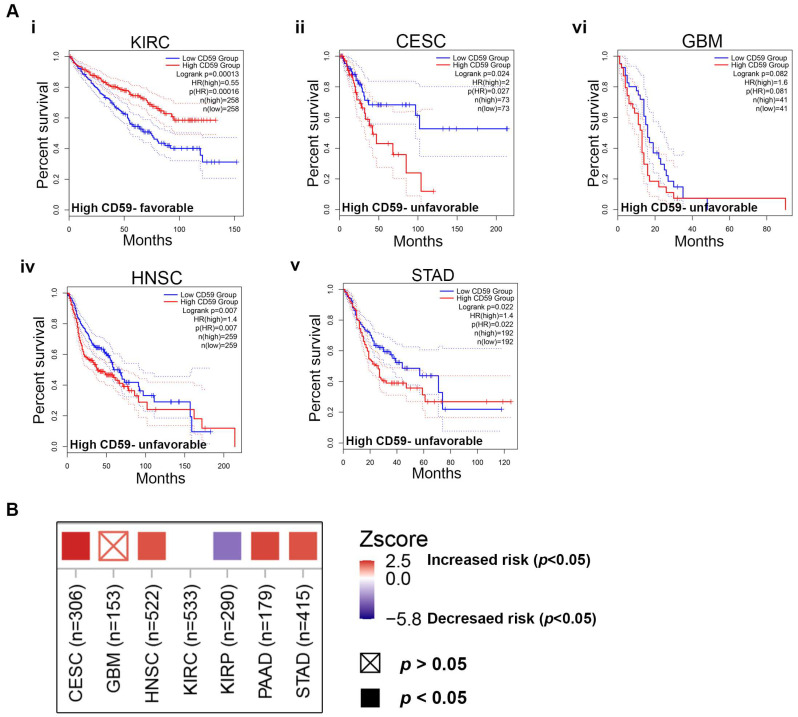
Prognostic analysis of CD59 in cancer. (**Ai**–**v**) Analysis of CD59 expression and overall survival (OS) using Kaplan–Meier in KIRC (**i**), CESC (**ii**), GBM (**iii**), HNSC (**iv**), and STAD (**v**) using the GEPIA 2 dataset. (**B**) Analysis of clinical relevance of CD59 expression across various cancer types using Timer 2.0 analytical tool.

This study focuses on cancers with elevated CD59 mRNA and protein expression, analyzing the correlation between CD59 levels and survival outcomes. The analysis was performed for five cancers with high CD59 expression using the TCGA database from GEPEA 2, focusing on overall survival (OS). The Kaplan–Meier curve (Figure 3A) showed that CD59 overexpression was linked to a poor prognosis in several cancers, including CESC, GBM, HNSC, and STAD, but was associated with a favorable prognosis in patients with KIRC (Figure 3A(i–v)). These cancers show poor prognostic outcomes in which CESC, HNSC, and STAD have significant *p* values (*p* < 0.05) and GBM has *p* = 0.071, respectively. Disease-free survival (DSS) data, along with the OS results, indicate that patients with KIRC have longer DSS rates, while those with CESC, GBM, HNSC, PAAD, and STAD along with elevated CD59 expression showed shorter DSS rates (Figure S2). The Timer 2.0 gene outcome module provides the clinical relevance of gene expression across various cancer types. The heatmap shown in Figure 3B indicates significantly high risk in CESC, HNSC, PAAD, and STAD and moderate risk in GBM, while KIRC and KIRP indicate low risk with high CD59 expression. These data suggest that elevated levels of CD59 can lead to varying outcomes, indicating the potential involvement of additional pathways.

### 3.4. Correlation Between Treg and MDSC with CD59 Expression Leads to Immune Suppression

**Figure 4:**
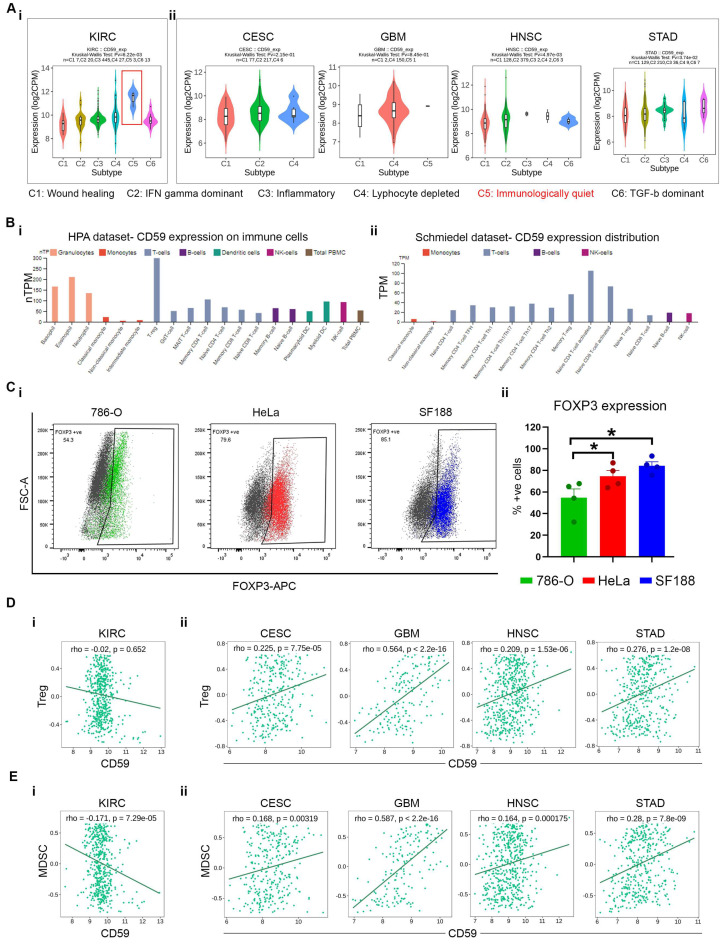
Correlation between CD59 expression and Treg and MDSC. (**A**) Immune subtype analysis of (**i**) KIRC, (**ii**) CESC, GBM, HNSC, and STAD using TISIDB. (**B**) expression and distribution of CD59 on immune cells by the HPA dataset (**Bi**) and Schmiedel database (**Bii**). (**C**) Flow cytometry analysis (Ci) with quantification (Cii) of FOXP3 intracellular expression on 786-O, HeLa, and SF188 cell lines. Correlation between CD59 and Treg cells (**D**) and MDSC cells (**E**) in (**i**) KIRC, (**ii**) CESC, GBM, HNSC, and STAD using TISIDB. Data represents a minimum of three independent experiments, where * indicates *p* ≤ 0.05, *t*-test.

Clinically significant cancers with high CD59 expression were further examined for immune subtype analysis using the TISIDB database, a central resource for studying tumor-immune system interactions. The TISIDB immune subtype analysis shows that favorable cancers like KIRC are immunologically quiet (Figure 4A(i)), while CESC, GBM, HNSC, and STAD are characterized by lymphocyte depletion and/or TGFβ dominance (Figure 4A(ii)). These findings suggest distinct immune environments between high CD59-expressing cancers with favorable versus unfavorable clinical outcomes. Analysis of CD59 expression on immune cells using the HPA and Schmiedel datasets reveals that CD59 is highly expressed on Treg cells, as well as naïve CD4+ and CD8+ T cells (Figure 4B(i,ii)). Expression of FOXP3 in tumors recruits Treg cells and immune suppression in the TME [9]. Flow cytometry analysis demonstrates that 786-O expresses low positivity (~45%) (Figure 4C(i,ii)). On the contrary, HeLa and SF188 show a significantly higher expression of FOXP3 (~73% and 93%, respectively) (Figure 4C(ii)). Furthermore, Qing Li et al. showed that CD59^−/−^ Treg cells exhibit greater complement-mediated injury than WT Teg cells [10]. To assess CD59’s role in the Treg population within cancers, we performed a correlation analysis between CD59 and Tregs across various cancers. The data indicate a negative correlation between CD59 and Tregs in KIRC, while CESC, GBM, HNSC, and STAD show a significant positive correlation (*p* < 0.05) (Figure 4D(i,ii)). This suggests that immunologically quiet cancers like KIRC have a less Treg-mediated immunosuppressive tumor microenvironment than CESC, GBM, HNSC, and STAD. In the presence of TGFβ1, Tregs regulate MDSC function [11], and CESC, GBM, HNSC, and STAD express high levels of TGFβ (Figure 4A). We further analyzed the correlation between CD59 and MDSCs in these cancers. In KIRC, CD59 and MDSC expression is significantly negatively correlated, while CESC, GBM, HNSC, and STAD show a significant positive correlation (Figure 4E(i,ii)). This further supports the evidence of a highly immunosuppressive tumor microenvironment in CESC, GBM, HNSC, and STAD compared to KIRC.

### 3.5. Association Between CD59 and Tumor-Associated M2 Macrophage Enhances Immune Suppression

**Figure 5:**
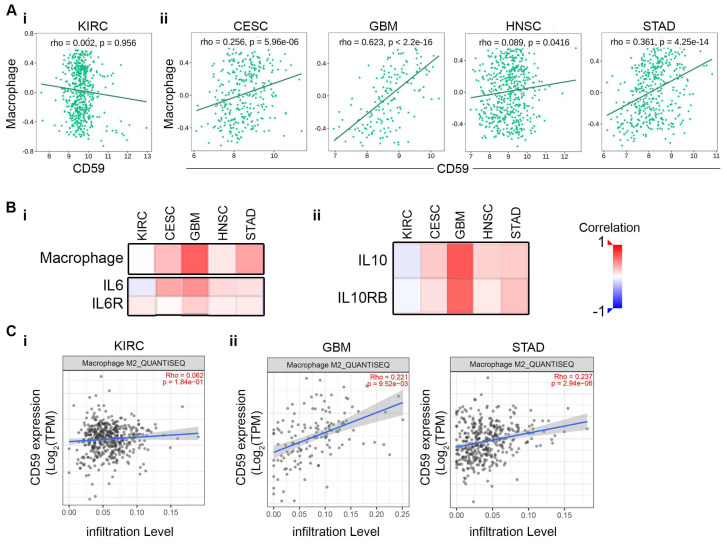
Correlation between CD59 expression and TAM and immune-infiltrating M2 macrophage: (**A**) Spearman correlation between CD59 and macrophage in (**i**) KIRC, (**ii**) CESC, GBM, HNSC, and STAD. (**B**) Heatmap of correlation between CD59 and (**i**) macrophage, IL6, IL6R, and (**ii**) IL10, and IL10RB in KIRC, CESC, GBM, HNSC, and STAD. (**C**) Scattered plot of relationship between M2 macrophage infiltration and CD59 expression in (**i**) KIRC, and (**ii**) GBM, and STAD using Timer 2.0 analytical tool.

Tumor-associated macrophages (TAMs) are versatile immune cells that promote a variety of malignant behaviors. Zhang et al. reported a correlation between TAM infiltration and CD59 expression in pancreatic cancer, noting that higher TAM infiltration was associated with higher CD59 levels, resulting in poorer patient survival [12]. Additionally, pancreatic cancer-educated macrophages could protect cancer cells from complement-mediated cytotoxicity by up-regulating CD59 in an IL6/STAT3-dependent manner [12]. Furthermore, Koch N. et al. found that IL10, a potent immunosuppressive anti-inflammatory cytokine, upregulates CD59 expression [13] and promotes M2 macrophage polarization [14]. To explore similar mechanisms in other cancers, we conducted a correlation analysis of CD59 with macrophage, IL10, IL10RB, IL6, and IL6R on KIRC, CESC, GBM, HNSC, and STAD. TISIDB analysis reveals a non-significant negative correlation between CD59 and macrophage in KIRC, while CESC, GBM, HNSC, and STAD show a significant positive correlation (as indicated by a rho value from 0.256 to 0.623 and a *p* value from 0.00000596 and below) (Figure 5A(i,ii)). Spearman correlation analysis further indicates a strong association between CD59 and macrophage, IL6, and IL6R in CESC, GBM, HNSC, and STAD, but no significant correlation in KIRC (Figure 5B(i)). Positive correlations were also observed between CD59 and IL10, IL10RB expressions in CESC, GBM, HNSC, and STAD cancer (Figure 5B(ii)), suggesting increased infiltration of the M2 macrophage in the TME of CESC, GBM, HNSC, and STAD cancers. Timer 2.0 analysis confirmed significantly higher CD59-expressing M2 macrophage infiltration in GBM and STAD compared to KIRC (Figure 5C(i,ii)). These results provide insights into the difference between CD59-mediated M2 macrophage infiltration and the immunosuppressive TME in CESC, GBM, HNSC, and STAD.

**Figure 6:**
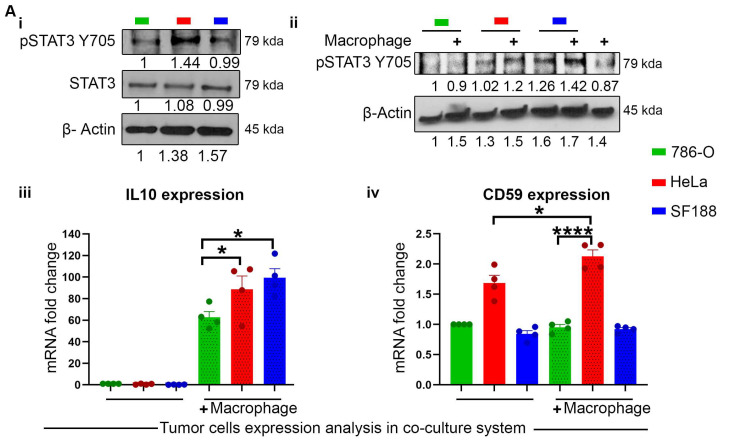

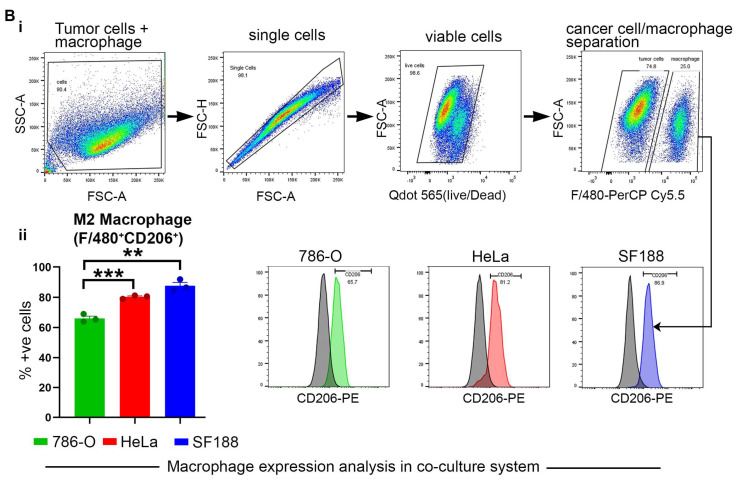
Co-culture of tumor cells with macrophage increases infiltrating M2 macrophage: (**A**) Analysis of tumor cells in the co-culture system. (**Ai**) Basic expression of phosphorylated STAT3 (Y705) in tumor cells. (**Aii**) Expression of pSTAT3 (Y705) in tumor cells in the presence or absence of macrophage. (**Aiii**) mRNA expression of IL10 in tumor cells in the presence or absence of macrophage. (**Aiv**) mRNA expression of CD59 in tumor cells in the presence or absence of macrophage. (**B**) Analysis of macrophage in the co-culture system. (**Bi**) Gating strategy to identify M2 macrophage. (**Bii**) Percentage of M2 macrophage in 786-O, HeLa, and SF188. Data represents a minimum of three independent experiments, where * indicates *p* ≤ 0.05, ** indicates *p* ≤ 0.01, *** indicates *p* ≤ 0.001, **** indicates *p* ≤ 0.0001, *t*-test.

To further confirm the impact of tumor cells on the macrophage, or vice versa, and the meta-analysis data, we performed a co-culture experiment with mouse macrophage (MΦ) and human cancer cells (786-O, HeLa, and SF188) and assessed the polarization status of macrophage. After 48 h of co-culture, Hela and SF188 cells showed increased activation and phosphorylation of STAT3 upon macrophage co-culture, while 786-O cells did not (Figure 6A(ii)). Additionally, HeLa and SF188 exhibit higher basal STAT3 activation (Figure 6A(i)). The phosphorylation of STAT3 is known to be mediated by IL6 or IL10 [12,13]. To confirm this mechanism, we analyzed the mRNA expression of IL10 (Figure 6A(iii)). Co-culture with MΦ led to elevated IL10 expression (Figure 6A(iii)), with significantly higher levels in HeLa and SF188 compared to 7860-O (Figure 6A(iii)). Increased IL10 expression and STAT3 phosphorylation were associated with higher CD59 expression in HeLa cells (Figure 6A(iv)).

Additionally, Co-cultured mouse macrophages (MΦ) are subjected to flow cytometry analysis using anti-mouse PerCP Cy5.5-F/480 and anti-mouse PE-CD206 to check M2 polarization status. M2 macrophages, identified by F/480^+^CD206^+^ (Figure 6B(i) gating strategy) were significantly more prevalent in HeLa and SF188 compared to 786-O (Figure 6B(ii)), indicating the establishment of an immune-suppressive TME in HeLa and SF188. These immune differences in the TME play a crucial role in deciding their predictive clinical outcomes.

### 3.6. TGFβ1 Co-Expression with CD59 Inhibits T Cell-Mediated Cytotoxicity

**Figure 7:**
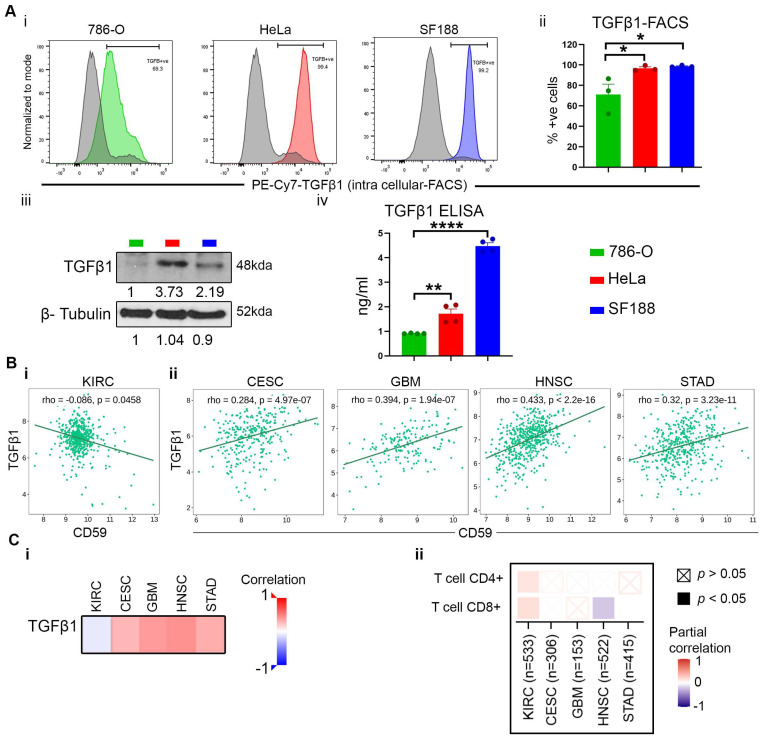
TGF-β-mediated immune suppression in CESC, GBM, HNSC, and STAD. (**A**) Expression analysis of TGFβ1. (**Ai**,**ii**) Flow cytometry analysis of TGF-β expression on 786-O, HeLa, and SF188 cell line. (**Aiii**) Immunoblotting of TGFβ1 and (**Aiv**) ELISA of secreted TGFβ1. (**B**) Spearman correlation analysis of TGF-β and CD59 expression in (**i**) KIRC, (**ii**) CESC, GBM, HNSC, and STAD and supported by (**Ci**) heat map of TGF-β and CD59 expression in KIRC, CESC, GBM, HNSC, and STAD using TISIDB. (**Cii**) Correlation between CD4+ and CD8+ T cell immune infiltration and CD59 expression in multiple cancers using Timer 2.0 analytical tool. β-tubulin (control) for Figure 7A(iii) (786-O, HeLa, and SF188) are similar to Figure 2B. Data represents a minimum of three independent experiments, where * indicates *p* ≤ 0.05, ** indicates *p* ≤ 0.01, **** indicates *p* ≤ 0.0001, *t*-test.

Transforming growth factor β1 (TGFβ1) is a multifaceted cytokine that plays a role in both immune suppression and inflammation [15]. Recent research has demonstrated that TGFβ1 plays a role in immune suppression across various cancers [16]. Immune subtype analysis characterizes these cancers to be TGFβ-dominant cancers, as shown in Figure 3A(i,ii). Goswami et al. showed a significant upregulation of CD59 expression on cell surfaces following epithelial-mesenchymal transition (EMT) induced by TGFβ [17]. This finding prompted us to assess TGFβ1 expression on in-house cell lines 786-O, HeLa, and SF188. Flow cytometry analysis of intracellular staining for TGFβ1 revealed a high level of TGFβ1 in HeLa and SF188 (~99% in both) (Figure 6A(i,ii)), with moderate levels observed in 786-O (~60%) (Figure 7A(i,ii)). These findings were corroborated by immunoblotting of same cell lines (Figure 7A(iii)). Secretion of TGFβ1 from tumor cells contributes to an immune suppressive TME by recruiting MDSCs and inhibiting cytotoxic T cells [18]. Therefore, we performed enzyme-linked immunosorbent assay (ELISA), revealing significantly higher levels of secreted TGFβ1 in HeLa and SF188 compared to 786-O (Figure 7A(iv)). This finding is consistent with the TCGA database of TGFβ1-dominant cancer. Furthermore, Spearman correlations between TGFβ1 and CD59 show a significant positive correlation in CESC, GBM, HNSC, and STAD cancer, whereas a negative correlation is observed in KIRC (Figure 7B(i,ii)), as further illustrated by a heat map of TGFβ1 and CD59 correlation (Figure 7C(i)). TGFβ is known to regulate homeostasis of the naïve T cells and suppress cytotoxic T lymphocytes [19]. We investigated the correlation between CD59 expression and the infiltration levels of CD4^+^ T cells and CD8^+^ T cells. The Timer 2.0 dataset reveals a significant positive correlation between CD59 expression and both CD4^+^ and CD8^+^ T cell infiltration exclusively in KIRC (Figure 7C(ii)). These data reinforce the notion that T cell-mediated cytotoxicity is inhibited in CESC, GBM, HNSC, and STAD cancer but not in KIRC.

## 4. Discussion

The high expression of CD59 on cancer cells facilities their evasion from CDC, leading to tumor progression. Elevated CD59 levels are associated with a worse prognosis in several cancers, including colorectal [20], prostatic [21], ovarian [22], and lung [23] cancers. Our study also demonstrates that high CD59 expression in CESC, GBM, HNSC, and STAD are correlated with a poor prognosis. In contrast, KIRC is linked to better outcomes with high CD59 expression. This prompted us to investigate how different immune environments within the TME interact with CD59 to influence the outcome. Li et al. demonstrated that half of the T reg cells express CD59, which is critical for maintaining immune homeostasis, and the knockdown of CD59 (CD59^−/−^) on Treg cells increases their susceptibility to complement-mediated injury [10]. This indicates that CD59 expression plays an integral role in Treg-mediated immune suppression. Our analysis reveals that the negative correlation between CD59 and Treg contributes to a less immune suppressive TME in KIRC, unlike the positive correlation in CESC, GBM, HNSC, and STAD cancers. This might be due to differences in CD59 expression on Treg cells within the TME and differences in the expression of FOXP3 among these tumors. Moreover, Treg cells can enhance MDSC function and modulate their differentiation via TGF-β. In TGFβ-dominant cancers like CESC, GBM, HNSC, and STAD, MDSCs are positively correlated with CD59, contributing to increased immune suppression.

TAMs play a pivotal role in tumor progression through multiple mechanisms, including enhanced angiogenesis [24], stemness [25], chemotherapy resistance [26], and suppression of anti-tumor immunity [27]. Zhang et al. showed a positive correlation between CD59 and TAM in clinical samples, leading to a worse prognosis in PAAD [12]. We observed similar correlations in CESC, GBM, HNSC, and STAD cancers, where a strong association between CD59 and TAM is linked to worse prognosis through IL10/STAT3-dependent pathways. However, in KIRC, no significant correlation was observed. Additionally, IL10, which is essential for the polarization of macrophages to an M2-like phenotype [14], also positively correlates with CD59 expression in CESC, GBM, HNSC, and STAD. To prove this hypothesis, co-culture experiments of tumor cells and MΦ provided robust evidence that CESC and GBM can polarize macrophage to a M2 state to create an immune suppressive TME by upregulating IL10 and CD59. M2 polarization is known to be pro-tumorigenic, and both GBM and STAD exhibit high M2 TAM infiltration.

TGFβ plays a complex role in the TME, acting as both a tumor suppressor in normal cells and early-stage cancer by inducing cell cycle arrest [28,29] and activating the SMAD-dependent apoptosis pathway [30]. However, in advanced cancer, it promotes tumor progression through EMT, immune evasion, angiogenesis and ECM remodeling [31] Goswami et al. [17] demonstrated that TGFβ1 induces CD59 expression in lung adenocarcinoma cells, which leads to resistance to CDC. Moreover, the induction of CD59 is Smad3-dependent. Our in vitro data suggests that HeLa and SF188 cells representing CESC and GBM, respectively, express significantly higher levels of TGFβ compared to 786-O cells, which represent KIRC. These data confirm that these cancers are TGF-β-dominant, unlike KIRC. Correlation analysis of CD59 and TGFβ shows a positive correlation in these cancers, but not in KIRC. TGF-β partially impairs cytotoxic T cell activity [32], and our data show that only KIRC has CD4^+^ and CD8^+^ T cell infiltration, whereas others have TGF-β-mediated inhibition of cytotoxic T cell infiltration.

**Figure 8:**
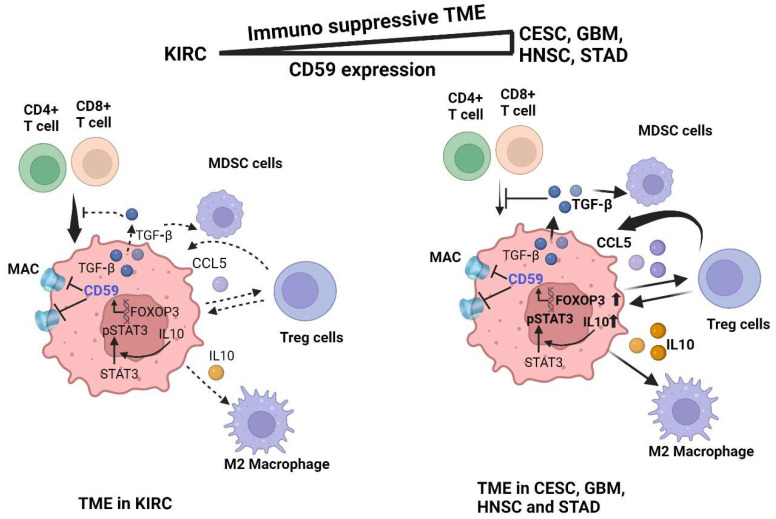
Diagram of CD59-mediated immune suppression in CESC, GBM, HNSC, and STAD. CESC, GBM, HNSC, and STAD have high amounts of FOXP3, IL10, TGFβ1, and pSTAT3, leading to increased CD59 transcription and the recruitment of immune suppressive cells such as MDSC, Treg, and TAM in the TME.

In this study, we have demonstrated that the correlation between immune cells in the TME and CD59 plays a decisive role in predicting prognosis. Immunologically quiet cancer such as KIRC, where CD59 has less interaction with immune suppressive cells such as Treg, MDSC, and TAM, predicts favorable outcomes. Contrary to this, CESC, GBM, HNSC, and STAD express high amounts of FOXP3, IL10, TGFβ, and phosphorylated STAT3, together with CD59, leading to recruitment of Immune suppressive cells, resulting in worse prognosis (Figure 8).

Furthermore, CD59 is known to have a complement-independent role in T cell activation and NK cell-mediated cytotoxicity [33]. This aspect warrants investigation to better understand how CD59 modulates the immune response in different cancers. Additionally, TGFβ-dominant cancers such as CESC, GBM, HNSC, and STAD may use CD59 as an immune escape mechanism by blocking MAC formation and increasing the population of Treg, MDSC, and TAM. Its association with immune cells across various cancer models reveals a complex relationship within the TME that shapes tumor progression. Our correlation analysis of CD59 emphasizes the importance of understanding its interactions with other immune components across different tumor models, as these dynamics can affect outcomes. Since CD59 is expressed in all cells, including both tumor and immune cells, solely silencing or overexpressing it in tumor cells may not yield predictive results. Targeting CD59 on both immune and tumor cells could potentially offer a novel therapeutic strategy for these cancers.

## 5. Conclusions

Overall, through a comprehensive analysis of CD59 expression across various cancers, we systematically evaluated its prognostic significance and its relationship with immune cells. These findings offer a theoretical framework and mechanisms using cell lines to understand the molecular, biological, and clinical aspects of cancers involving CD59 expression. Our results indicate that elevated CD59 levels in many cancers contribute to the inhibition of MAC-mediated cytotoxicity, correlating with poor outcome; however, KIRC has the opposite trend with favorable outcomes. Further investigation has shown that CD59-mediated outcomes are affected by the correlation between CD59 and immune-suppressive cell types such as Treg, MDSC, TAM, and TGF-β. This correlation appears to predict adverse outcomes in cancers like CESC, GBM, HNSC, and STAD versus favorable outcomes in KIRC. Despite these insights, the study mainly relies on publicly available databases, highlighting the need for additional animal research to elucidate the role of CD59 and its interactions with immune cells in cancer.

## Figures and Tables

**Table 1:** Biological pathways related to the proteins involved in CD59 networks based on KEGG pathways (based on STRING database).

Pathway	Description	Protein Gene
hsa04130	SNARE interactions in vesicular transport	SNAP29, BETI, YKT6, GOSR2, STX17, VAMP7, VAMP8, STX5, STX18, SEC22B
hsa04610	Complement and coagulation cascades	C5, C8G, C3, C9, C6, CD46, C7, C5AR1, C8A, CD55, C8B, CD59
hsa05322	Systemic lupus erythematosus	C5, C8G, C3, C9, C6, C7, CD28, C8A, FCGR3A, C8B
hsa0563	Glycosylphosphatidylinositol (GPI) anchor biosynthesis	PIGQ, PIGH, PIGC, PIGA
hsa0520	Prion disease	C5, C3, C5AR1, FCGR3A
hsa04650	Natural killer cell-mediated cytotoxicity	FCER1G, NCR1, CD247, FCGR3A, CD48
hsa05150	Staphylococcus aureus infection	C5, C3, C5AR1, FCGR3A
hsa05146	Amoebiasis	C8G, C9, C8A, C8B
hsa04140	Autophagy animal	SNAP29, ATG14, STX17, VAMP8
hsa04145	Phagosome	C3, STX18, FCGR3A, SEC22B

**Table 2:** List of cancers and CD59 expression with significance.

Cancer Type	Protein Expression	*p* Value	Significance	Condition
Breast cancer	Moderate/Low	0.021	Not prognostic	
Cervical cancer	High	0.000038	Prognostic,	Unfavorable
Colorectal cancer	Low	0.074	Not prognostic	
Endometrial cancer	Moderate/Low	0.011	Not prognostic	
Glioma	Moderate to High	0.026	May be prognostic	Unfavorable
Head and neck cancer	High	0.000031	Prognostic	Unfavorable
Liver cancer	Moderate	0.056	Not prognostic	
Lung adenocarcinoma	Moderate/Low	0.16	Not prognostic	
Lung squamous cell carcinoma	Moderate/Low	0.0078	Not prognostic	
Melanoma cancer	High	0.045	Not prognostic	
Ovarian cancer	Low	0.30	Not prognostic	
Pancreatic cancer	High	0.000029	Prognostic	Unfavorable
Prostate cancer	Low	0.017	Not prognostic	
Renal cancer	High	0.00000000014	Prognostic	Favorable
Stomach cancer	Moderate	0.00090	Prognostic	Unfavorable
Testis cancer	Low	0.13	Not prognostic	
Thyroid cancer	High	0.010	Not prognostic	Favorable
Urothelial cancer	Moderate/Low	0.31	Not prognostic	

## Data Availability

Data are contained within the article and Supplementary Materials.

## Supplementary Materials

The following supporting information can be downloaded at: https://www.mdpi.com/article/10.3390/cancers16213699/s1, Figure S1: Gene ontology and pathway Enrichment Analysis. (A) string analysis of CD59 to identify significantly related genes (B) the GO analysis of CD59 in three aspects: cellular composition (CC), biological process (BP), and molecular function (MF). (C) Top 10 KEGG pathways regulated by CD59. Figure S2: Analysis of CD59 expression and Disease-free Survival (DSS) using Kaplan–Meier in KIRC, CESC, GBM, HNSC and STAD using GAPIA2 analytical tool.
