# Supplementary material for: Deciphering CD59: Unveiling Its Role in Immune Microenvironment and Prognostic Significance
Source: Cancers (Basel). 2024 Nov 1;16(21):3699. doi: 10.3390/cancers16213699 (PMC11545456; doi:10.3390/cancers16213699)

Supplemental material

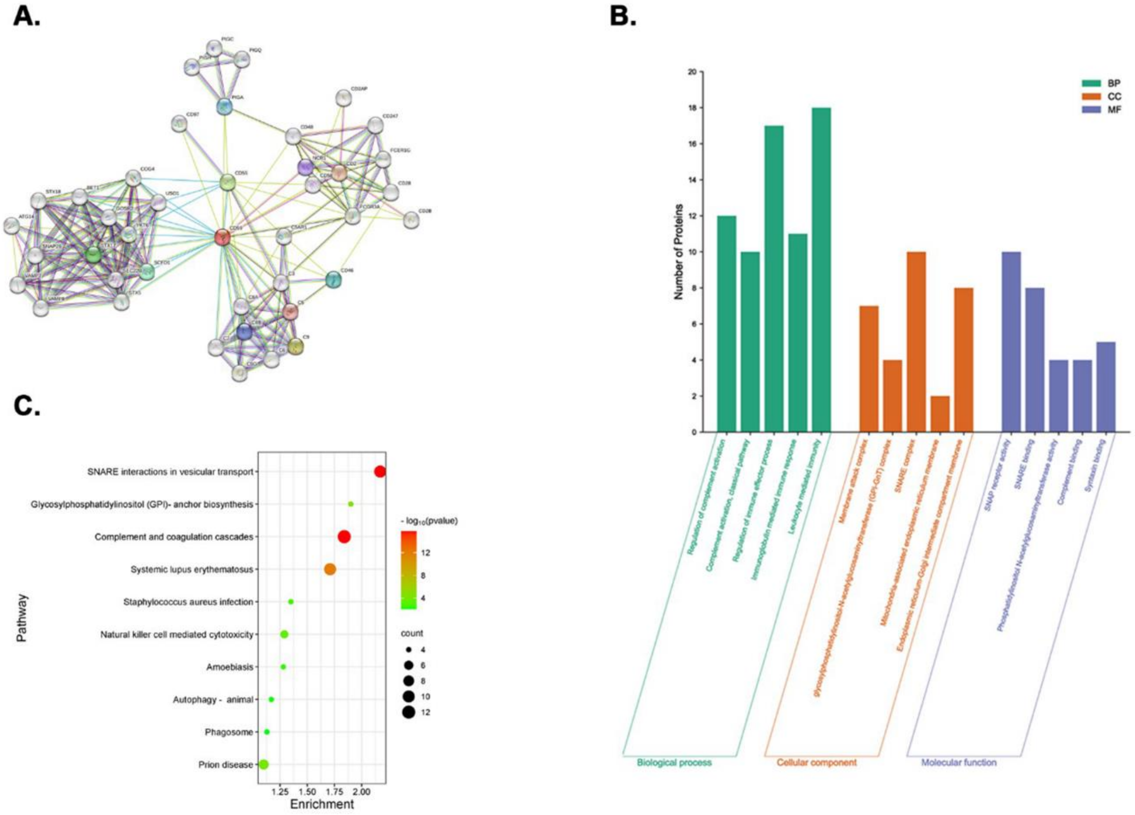

Fig. 1S Gene ontology and pathway Enrichment Analysis. (A) string analysis of CD59 to identify significantly related genes (B) the GO analysis of CD59 in three aspects: cellular composition (CC), biological process (BP), and molecular function (MF). (C) Top 10 KEGG pathways regulated by CD59.

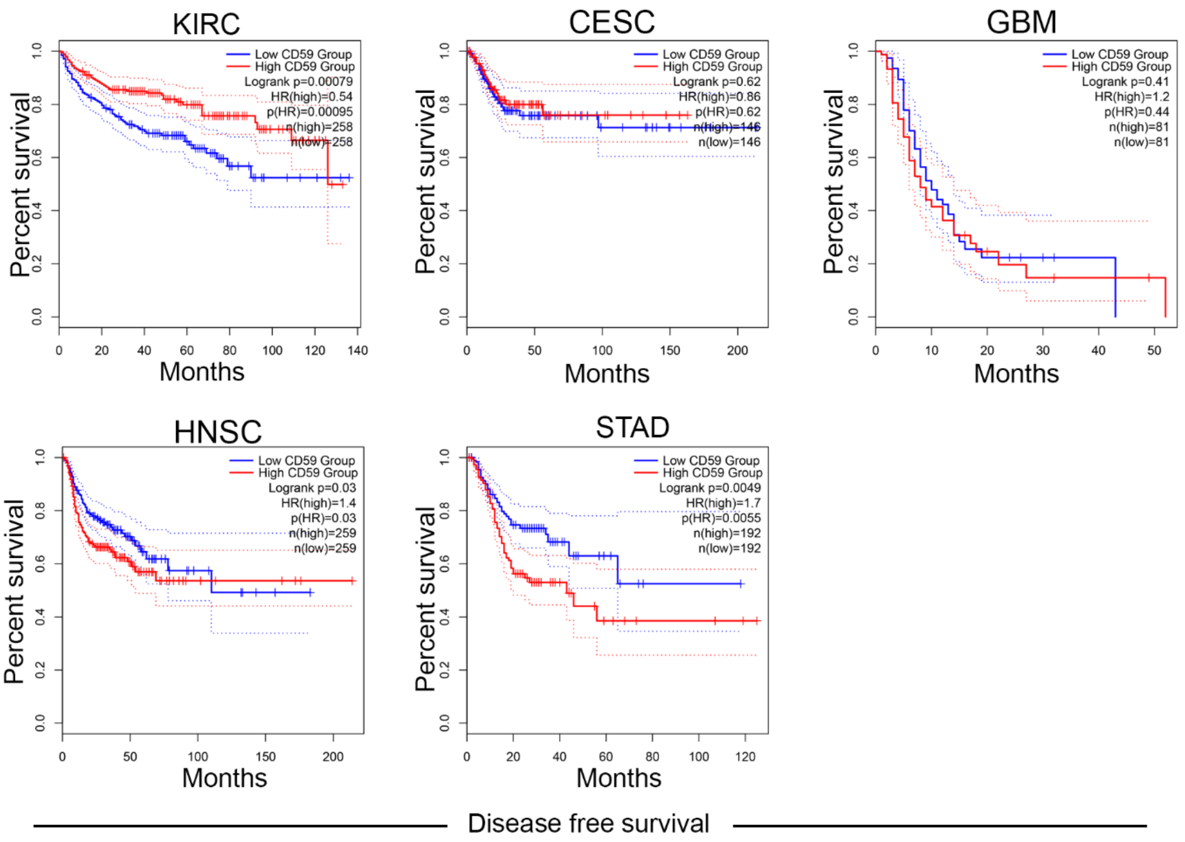

Fig. 2S Analysis of CD59 expression and Disease-free Survival (DSS) using Kaplan–Meier in KIRC, CESC, GBM, HNSC and STAD using GAPIA2 analytical tool.

Raw western blots and densitometry.

Fig. 2B & 7Aiii

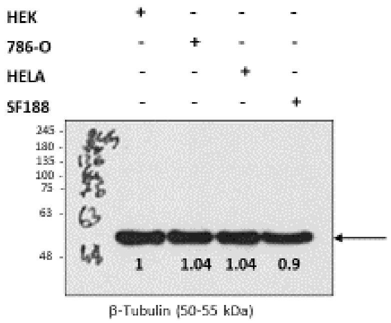

Fig. 2B

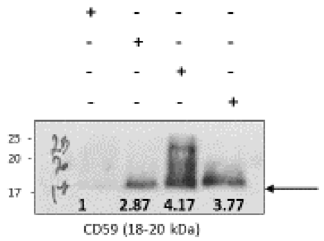

Fig. 7Aiii

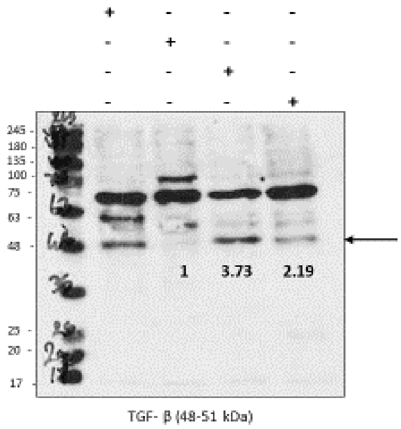

Fig. 6Ai

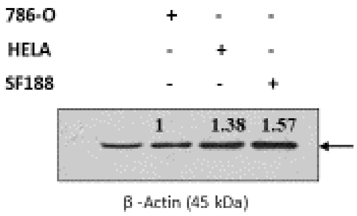

Fig. 6Ai

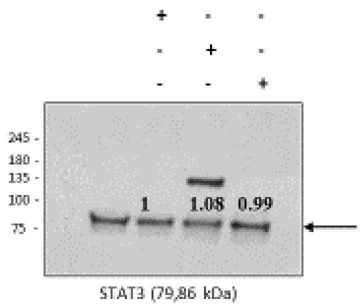

Fig. 6Ai

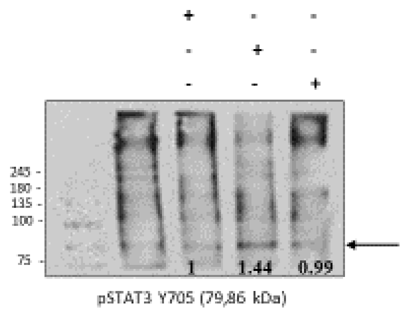

Fig. 6Aii

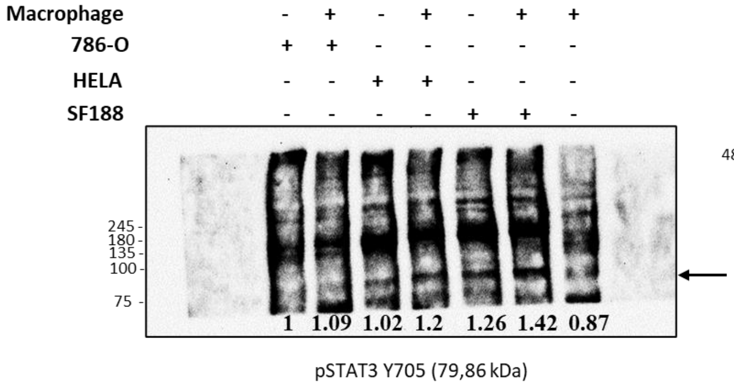

Fig. 6Aii

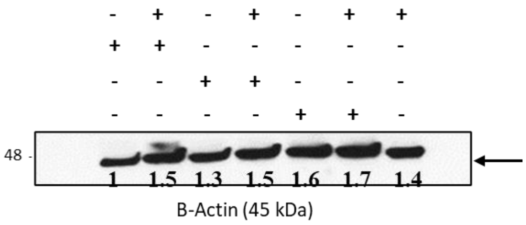

Supplement: Supplementary file 1 [file cancers-16-03699-s001.zip › cancers-3239290-supplementary.pdf]
